# Penta­carbon­yl{3-[(2*S*)-1-methyl­pyrrolidin-2-yl]pyridine}tungsten(0)

**DOI:** 10.1107/S1600536810010974

**Published:** 2010-03-31

**Authors:** Martin O. Onani, Roger A. Lalancette, Naftali T. Muriithi, Eunice A. Nyawade, Boitumelo V. Kgarebe

**Affiliations:** a University of the Western Cape, Cape Town, Bellville 7535, South Africa; b Department of Chemistry, Rutgers, the State University of New Jersey, 73 Warren St, Newark, NJ 07102, USA; c Department of Chemistry, Kenyatta University, PO Box 43844-00100, Nairobi, Kenya

## Abstract

The title compound, [W(C_10_H_14_N_2_)(CO)_5_], contains five carbonyl ligands and a nicotine ligand in an octa­hedral arrangement around the tungsten atom. The metal atom shows *cis* angles in the range 87.30 (16)–94.2 (2)°, and *trans* angles between 175.2 (2) and 178.1 (4)°. The W—CO bond *trans* to the pyridine N atom [1.987 (6) Å] is noticeably shorter than the others, which range between 2.036 (3) and 2.064 (3) Å, possibly due to the well-known *trans* effect. The distance between the W atom and the pyridine N atom is 2.278 (4) Å.

## Related literature

Attempts to understand and mimic the nature of nitro­gen fixation have led to studies on the responsible enzyme, nitro­genase (Jennings, 1991 ▶; Schrock, 2006 ▶). As part of this work, we have investigated the reactions of the precursor tungsten halogenidocarbonyl derivative, [W(CO)_4_ *X*_2_]_2_, with nitro­gen bases. For possible reaction mechanisms, see: Abel *et al.* (1963 ▶); Baker (1998 ▶); Heyns & Buchholtz (1976 ▶); Tripathi & Srivasatva (1970 ▶). For the preparation of tungsten dichlorido­tetracarbonyl [W(CO)_4_Cl_2_], see: Colton & Tomkins (1966 ▶).
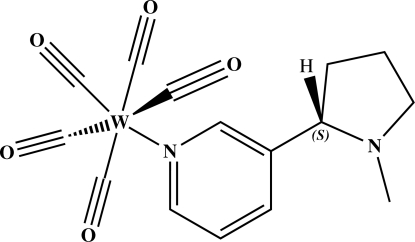


## Experimental

### 

#### Crystal data

[W(C_10_H_14_N_2_)(CO)_5_]
*M* *_r_* = 486.13
Monoclinic, 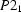

*a* = 6.6303 (1) Å
*b* = 10.6720 (2) Å
*c* = 11.6748 (2) Å
β = 96.636 (1)°
*V* = 820.56 (2) Å^3^
*Z* = 2
Cu *K*α radiation
μ = 13.29 mm^−1^
*T* = 100 K
0.28 × 0.25 × 0.19 mm

#### Data collection

Bruker SMART CCD APEXII area-detector diffractometer
Absorption correction: numerical (*SADABS*; Sheldrick, 2008*a* ▶) *T*_min_ = 0.119, *T*_max_ = 0.185
6704 measured reflections
2676 independent reflections
2673 reflections with *I* > 2σ(*I*)
*R*_int_ = 0.020

#### Refinement

*R*[*F* ^2^ > 2σ(*F* ^2^)] = 0.014
*wR*(*F* ^2^) = 0.033
*S* = 1.13
2676 reflections
210 parameters
1 restraint
H-atom parameters constrained
Δρ_max_ = 0.44 e Å^−3^
Δρ_min_ = −0.53 e Å^−3^
Absolute structure: Flack (1983 ▶), 1225 Friedel pairs
Flack parameter: 0.041 (9)

### 

Data collection: *APEX2* (Bruker, 2006 ▶); cell refinement: *SAINT* (Bruker, 2005 ▶); data reduction: *SAINT*; program(s) used to solve structure: *SHELXTL* (Sheldrick, 2008*b* ▶); program(s) used to refine structure: *SHELXTL*; molecular graphics: *SHELXTL*; software used to prepare material for publication: *SHELXTL*.

## Supplementary Material

Crystal structure: contains datablocks I, global. DOI: 10.1107/S1600536810010974/ez2203sup1.cif

Structure factors: contains datablocks I. DOI: 10.1107/S1600536810010974/ez2203Isup2.hkl

Additional supplementary materials:  crystallographic information; 3D view; checkCIF report

## Figures and Tables

**Fig. 1.**
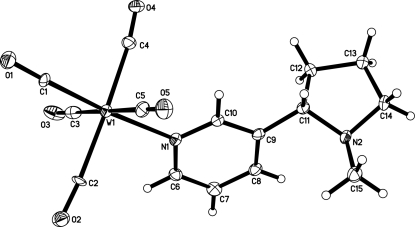
: The structure of the asymmetric unit of (I) with its numbering scheme. Displacement ellipsoids are drawn at the 40% probability level for non-H atoms.

## supplementary crystallographic information

### Comment

An effort to understand and mimic the nature of nitrogen fixation has led to wide studies on the responsible enzyme, nitrogenase (Jennings, 1991; Schrock, 2006). The focus of our study was to investigate the reactions of the precursor tungsten halocarbonyl derivative, [W(CO)_4_*X*_2_]_2_, with nitrogen bases to help contribute to this investigation. The compound nicotine (*L*), was reacted with the presupposed W(CO)_4_*X*_2_, with the hope of obtaining W(CO)_4_*L*_2_; however, the product which was confirmed by a single-crystal X-ray analysis was W(CO)_5_L, indicating that the compound was formed *via* a different route. It is thus envisaged that during the preparation of the precursor it is likely that such compounds as W(CO)_5_Cl^-^ [Abel *et al.* (1963); Baker (1998)] may be formed, which in turn react with a single molecule of the ligand, nicotine, forming the obtained product. There is less likelihood of a direct substitution of the carbonyl ligands (Tripathi & Srivasatva, 1970) or a redox mechanism (Heyns & Buchholtz, 1976) that would most likely lead to a disubstituted compound. The coordination of nicotine to tungsten, as observed, is *via* the imine nitrogen as shown in the crystal structure of the compound (See Figure 1).

The W metal centre has *cis* angles in the range 87.30 (16) to 94.2 (2)°, and *trans* angles between 175.2 (2)° and 178.1 (4)°. The W–CO bond *trans* to the nitrogen [1.987 (6) Å] is noticeably shorter than the others, which range between 2.036 (3) and 2.064 (3) Å, possibly due to the known *trans* effect. The distance between the W atom and the nicotine imine N is 2.278 (4) Å.

The infrared absorption bands of the compound show five absorption peaks at 2006.8(*m*), 1921.9(w), 1884.3(*s*), 1816.6(*m*) and 1760.9(*m*) cm^-1^. Also, there is a ~1ppm shift downfield in the positions of the alpha protons on the pyridine ring of the product in both the ^1^H and ^13^C NMR spectra compared to the reactants. The CO that is *trans* to the pyridinyl structure has the most significant shift upfield, possibly due to the *trans* effect. Only one peak is observed for the carbonyls in the ^13^C NMR spectrum, possibly due to shielding by the ring electrons leading to a slow decay, and since they are so close they appear identical.

### Experimental

The procedure by Colton and Tomkins (1966) and Schlenk techniques were used to prepare tungsten halocarbonyl [W(CO)_4_Cl_2_]. This halocarbonyl (13.13 mmol) was then reacted *in situ* with the base, nicotine (31.13 mmol), dissolved in freshly distilled methanol (20 ml) at -78 °C and the mixture left to warm to room temperature while stirring. The solution was stirred at room temperature for 14 h after which the solvent was removed under vacuum and the residue washed with portions of dry freshly distilled methanol and rinsed with diethylether. A yellow product was obtained in medium yield.

The crystal was grown at 4°C using a slow diffusion of dichloromethane over hexane for several days.

### Refinement

All H atoms for (I) were found in electron density difference maps. The methyl H atoms were put in ideally staggered positions with C—H distances of 0.98 Å and *U*_iso_(H) = 1.5*U*_eq_(C). The methylene, methine and phenyl Hs were placed in geometrically idealized positions and constrained to ride on their parent C atoms with C—H distances of 0.99, 1.00 and 0.95 Å, respectively, and *U*_iso_(H) = 1.2*U*_eq_(C).

### Crystal data

**Table:**

[W(C_10_H_14_N_2_)(CO)_5_]	*F*(000) = 464
*M**_r_* = 486.13	*D*_x_ = 1.968 Mg m^−^^3^
Monoclinic, *P*2_1_	Cu *K*α radiation, λ = 1.54178 Å
Hall symbol: P 2yb	Cell parameters from 6772 reflections
*a* = 6.6303 (1) Å	θ = 3.8–66.8°
*b* = 10.6720 (2) Å	µ = 13.29 mm^−^^1^
*c* = 11.6748 (2) Å	*T* = 100 K
β = 96.636 (1)°	Plate, yellow
*V* = 820.56 (2) Å^3^	0.28 × 0.25 × 0.19 mm
*Z* = 2	

### Data collection

**Table:**

Bruker SMART CCD APEXII area-detector diffractometer	2676 independent reflections
Radiation source: fine-focus sealed tube	2673 reflections with *I* > 2σ(*I*)
graphite	*R*_int_ = 0.020
φ and ω scans	θ_max_ = 66.8°, θ_min_ = 3.8°
Absorption correction: numerical (*SADABS*; Sheldrick, 2008a)	*h* = −7→7
*T*_min_ = 0.119, *T*_max_ = 0.185	*k* = −12→12
6704 measured reflections	*l* = −13→11

### Refinement

**Table:**

Refinement on *F*^2^	Hydrogen site location: inferred from neighbouring sites
Least-squares matrix: full	H-atom parameters constrained
*R*[*F*^2^ > 2σ(*F*^2^)] = 0.014	*w* = 1/[σ^2^(*F*_o_^2^) + (0.0071*P*)^2^] where *P* = (*F*_o_^2^ + 2*F*_c_^2^)/3
*wR*(*F*^2^) = 0.033	(Δ/σ)_max_ < 0.001
*S* = 1.13	Δρ_max_ = 0.44 e Å^−^^3^
2676 reflections	Δρ_min_ = −0.53 e Å^−^^3^
210 parameters	Extinction correction: *SHELXTL* (Sheldrick, 2008b), Fc^*^=kFc[1+0.001xFc^2^λ^3^/sin(2θ)]^-1/4^
1 restraint	Extinction coefficient: 0.00053 (7)
Primary atom site location: structure-invariant direct methods	Absolute structure: Flack (1983), 1225 Friedel pairs
Secondary atom site location: difference Fourier map	Flack parameter: 0.041 (9)

### Special details

**Table:**

Experimental. 'crystal mounted on a Cryoloop using Paratone-N'
Geometry. All esds (except the esd in the dihedral angle between two l.s. planes) are estimated using the full covariance matrix. The cell esds are taken into account individually in the estimation of esds in distances, angles and torsion angles; correlations between esds in cell parameters are only used when they are defined by crystal symmetry. An approximate (isotropic) treatment of cell esds is used for estimating esds involving l.s. planes.
Refinement. Refinement of *F*^2^ against ALL reflections. The weighted *R*-factor wR and goodness of fit *S* are based on *F*^2^, conventional *R*-factors *R* are based on *F*, with *F* set to zero for negative *F*^2^. The threshold expression of *F*^2^ > σ(*F*^2^) is used only for calculating *R*-factors(gt) etc. and is not relevant to the choice of reflections for refinement. *R*-factors based on *F*^2^ are statistically about twice as large as those based on *F*, and *R*- factors based on ALL data will be even larger.

### Fractional atomic coordinates and isotropic or equivalent isotropic displacement parameters (Å^2^)

**Table:**

	*x*	*y*	*z*	*U*_iso_*/*U*_eq_	
W1	0.975072 (14)	0.99294 (4)	0.798344 (8)	0.01869 (6)	
C1	1.1233 (8)	1.1176 (5)	0.9028 (5)	0.0212 (12)	
O1	1.2053 (6)	1.1868 (3)	0.9687 (3)	0.0307 (8)	
N1	0.7925 (6)	0.8443 (4)	0.6915 (4)	0.0218 (9)	
C2	0.8653 (6)	1.1332 (4)	0.6881 (4)	0.0206 (10)	
O2	0.8203 (4)	1.2112 (3)	0.6253 (2)	0.0327 (6)	
N2	0.3021 (3)	0.5061 (4)	0.6952 (2)	0.0230 (6)	
C3	1.2185 (4)	0.9810 (7)	0.7074 (2)	0.0238 (9)	
O3	1.3582 (3)	0.9783 (5)	0.6594 (2)	0.0384 (8)	
C4	1.1080 (9)	0.8553 (5)	0.9047 (6)	0.0233 (12)	
O4	1.1972 (6)	0.7822 (3)	0.9596 (3)	0.0337 (8)	
C5	0.7301 (4)	1.0112 (5)	0.8913 (2)	0.0204 (11)	
O5	0.5952 (4)	1.0199 (2)	0.9427 (2)	0.0327 (8)	
C6	0.7783 (5)	0.8398 (3)	0.5751 (3)	0.0244 (7)	
H6	0.8463	0.9017	0.5355	0.029*	
C7	0.6690 (5)	0.7487 (3)	0.5122 (3)	0.0289 (7)	
H7	0.6611	0.7487	0.4304	0.035*	
C8	0.5703 (5)	0.6569 (3)	0.5682 (3)	0.0245 (7)	
H8	0.4963	0.5924	0.5258	0.029*	
C9	0.5815 (5)	0.6609 (3)	0.6876 (3)	0.0204 (6)	
C10	0.6930 (4)	0.7558 (3)	0.7446 (3)	0.0190 (6)	
H10	0.7001	0.7587	0.8263	0.023*	
C11	0.4868 (5)	0.5628 (3)	0.7575 (3)	0.0204 (6)	
H11	0.4530	0.6007	0.8312	0.025*	
C12	0.6199 (5)	0.4479 (3)	0.7839 (3)	0.0285 (8)	
H12A	0.7203	0.4626	0.8522	0.034*	
H12B	0.6931	0.4261	0.7174	0.034*	
C13	0.4704 (5)	0.3435 (4)	0.8078 (3)	0.0283 (8)	
H13A	0.4955	0.2666	0.7642	0.034*	
H13B	0.4821	0.3232	0.8911	0.034*	
C14	0.2606 (5)	0.3988 (3)	0.7666 (3)	0.0246 (7)	
H14A	0.1746	0.3365	0.7210	0.030*	
H14B	0.1908	0.4258	0.8329	0.030*	
C15	0.1310 (5)	0.5916 (4)	0.6783 (3)	0.0321 (8)	
H15A	0.0156	0.5494	0.6343	0.048*	
H15B	0.1687	0.6655	0.6359	0.048*	
H15C	0.0930	0.6174	0.7535	0.048*	

### Atomic displacement parameters (Å^2^)

**Table:**

	*U*^11^	*U*^22^	*U*^33^	*U*^12^	*U*^13^	*U*^23^
W1	0.01884 (8)	0.01807 (8)	0.01892 (8)	−0.00160 (9)	0.00110 (4)	−0.00046 (9)
C1	0.017 (2)	0.024 (3)	0.022 (3)	0.0024 (17)	0.0021 (17)	0.0015 (18)
O1	0.0287 (18)	0.0293 (18)	0.033 (2)	−0.0064 (14)	0.0007 (14)	−0.0016 (14)
N1	0.021 (2)	0.0222 (18)	0.0211 (18)	0.0006 (15)	−0.0020 (14)	−0.0023 (12)
C2	0.021 (2)	0.0141 (18)	0.025 (2)	0.0005 (17)	−0.0033 (17)	0.0001 (13)
O2	0.0357 (14)	0.0301 (15)	0.0304 (15)	0.0040 (12)	−0.0037 (11)	0.0013 (12)
N2	0.0172 (10)	0.0248 (18)	0.0262 (12)	−0.0042 (15)	−0.0002 (8)	0.0002 (15)
C3	0.0216 (12)	0.028 (3)	0.0223 (14)	0.0030 (19)	0.0068 (10)	0.0013 (18)
O3	0.0368 (12)	0.042 (2)	0.0386 (13)	0.0094 (16)	0.0119 (9)	0.0142 (15)
C4	0.024 (2)	0.018 (2)	0.026 (3)	−0.0011 (18)	−0.0001 (18)	0.0026 (17)
O4	0.042 (2)	0.0292 (17)	0.0269 (19)	−0.0006 (15)	−0.0089 (14)	0.0064 (14)
C5	0.0202 (12)	0.022 (3)	0.0200 (14)	−0.0013 (14)	0.0070 (10)	−0.0048 (14)
O5	0.0327 (12)	0.031 (3)	0.0349 (14)	−0.0026 (10)	0.0071 (9)	−0.0025 (9)
C6	0.0282 (17)	0.0266 (19)	0.0185 (19)	−0.0014 (14)	0.0029 (13)	0.0036 (14)
C7	0.0345 (19)	0.035 (2)	0.0170 (18)	−0.0022 (15)	0.0021 (13)	−0.0001 (13)
C8	0.0281 (17)	0.0268 (18)	0.0175 (18)	−0.0031 (14)	−0.0016 (12)	−0.0027 (12)
C9	0.0167 (14)	0.0233 (17)	0.0209 (18)	0.0029 (12)	0.0005 (11)	−0.0001 (12)
C10	0.0199 (15)	0.0220 (17)	0.0150 (16)	0.0017 (12)	0.0019 (11)	−0.0005 (11)
C11	0.0196 (16)	0.0228 (18)	0.0190 (18)	−0.0019 (14)	0.0025 (11)	−0.0026 (14)
C12	0.0234 (17)	0.0250 (18)	0.037 (2)	−0.0056 (13)	0.0013 (14)	0.0076 (12)
C13	0.0271 (19)	0.027 (2)	0.030 (2)	−0.0056 (13)	0.0005 (14)	0.0053 (13)
C14	0.0224 (16)	0.0218 (17)	0.0299 (19)	−0.0067 (13)	0.0039 (13)	−0.0018 (13)
C15	0.0243 (17)	0.0310 (19)	0.040 (2)	0.0035 (14)	0.0013 (14)	0.0027 (15)

### Geometric parameters (Å, °)

**Table:**

W1—C1	1.987 (6)	C7—H7	0.9500
W1—C3	2.035 (3)	C8—C9	1.388 (5)
W1—C2	2.052 (5)	C8—H8	0.9500
W1—C4	2.055 (6)	C9—C10	1.379 (5)
W1—C5	2.064 (3)	C9—C11	1.507 (5)
W1—N1	2.278 (4)	C10—H10	0.9500
C1—O1	1.156 (7)	C11—C12	1.522 (4)
N1—C10	1.344 (5)	C11—H11	1.0000
N1—C6	1.352 (6)	C12—C13	1.539 (5)
C2—O2	1.126 (6)	C12—H12A	0.9900
N2—C15	1.451 (5)	C12—H12B	0.9900
N2—C14	1.460 (5)	C13—C14	1.536 (4)
N2—C11	1.480 (4)	C13—H13A	0.9900
C3—O3	1.138 (4)	C13—H13B	0.9900
C4—O4	1.132 (7)	C14—H14A	0.9900
C5—O5	1.138 (4)	C14—H14B	0.9900
C6—C7	1.374 (5)	C15—H15A	0.9800
C6—H6	0.9500	C15—H15B	0.9800
C7—C8	1.384 (5)	C15—H15C	0.9800
			
C1—W1—C3	89.8 (2)	C10—C9—C11	118.9 (3)
C1—W1—C2	90.6 (3)	C8—C9—C11	123.0 (3)
C3—W1—C2	87.9 (2)	N1—C10—C9	124.0 (3)
C1—W1—C4	87.75 (12)	N1—C10—H10	118.0
C3—W1—C4	87.7 (2)	C9—C10—H10	118.0
C2—W1—C4	175.3 (2)	N2—C11—C9	113.0 (3)
C1—W1—C5	88.7 (2)	N2—C11—C12	101.4 (3)
C3—W1—C5	178.1 (3)	C9—C11—C12	113.7 (2)
C2—W1—C5	90.97 (18)	N2—C11—H11	109.5
C4—W1—C5	93.4 (2)	C9—C11—H11	109.5
C1—W1—N1	175.2 (2)	C12—C11—H11	109.5
C3—W1—N1	94.2 (2)	C11—C12—C13	104.5 (3)
C2—W1—N1	92.12 (11)	C11—C12—H12A	110.9
C4—W1—N1	89.8 (3)	C13—C12—H12A	110.9
C5—W1—N1	87.30 (16)	C11—C12—H12B	110.9
O1—C1—W1	176.2 (5)	C13—C12—H12B	110.9
C10—N1—C6	117.2 (4)	H12A—C12—H12B	108.9
C10—N1—W1	119.7 (3)	C14—C13—C12	104.1 (3)
C6—N1—W1	123.1 (3)	C14—C13—H13A	110.9
O2—C2—W1	174.5 (4)	C12—C13—H13A	110.9
C15—N2—C14	112.0 (2)	C14—C13—H13B	110.9
C15—N2—C11	113.5 (3)	C12—C13—H13B	110.9
C14—N2—C11	103.9 (3)	H13A—C13—H13B	109.0
O3—C3—W1	177.1 (6)	N2—C14—C13	104.9 (3)
O4—C4—W1	173.8 (5)	N2—C14—H14A	110.8
O5—C5—W1	179.3 (5)	C13—C14—H14A	110.8
N1—C6—C7	122.3 (3)	N2—C14—H14B	110.8
N1—C6—H6	118.9	C13—C14—H14B	110.8
C7—C6—H6	118.9	H14A—C14—H14B	108.8
C6—C7—C8	119.8 (3)	N2—C15—H15A	109.5
C6—C7—H7	120.1	N2—C15—H15B	109.5
C8—C7—H7	120.1	H15A—C15—H15B	109.5
C7—C8—C9	118.7 (3)	N2—C15—H15C	109.5
C7—C8—H8	120.7	H15A—C15—H15C	109.5
C9—C8—H8	120.7	H15B—C15—H15C	109.5
C10—C9—C8	118.1 (3)		
			
C3—W1—C1—O1	−139 (7)	C1—W1—C5—O5	−122 (22)
C2—W1—C1—O1	133 (7)	C3—W1—C5—O5	−159 (22)
C4—W1—C1—O1	−51 (7)	C2—W1—C5—O5	147 (22)
C5—W1—C1—O1	42 (7)	C4—W1—C5—O5	−35 (22)
N1—W1—C1—O1	8(9)	N1—W1—C5—O5	55 (22)
C1—W1—N1—C10	−11 (3)	C10—N1—C6—C7	−0.5 (6)
C3—W1—N1—C10	135.7 (3)	W1—N1—C6—C7	179.4 (3)
C2—W1—N1—C10	−136.2 (3)	N1—C6—C7—C8	−0.6 (6)
C4—W1—N1—C10	48.1 (3)	C6—C7—C8—C9	1.2 (5)
C5—W1—N1—C10	−45.3 (3)	C7—C8—C9—C10	−0.8 (5)
C1—W1—N1—C6	169 (2)	C7—C8—C9—C11	−177.5 (3)
C3—W1—N1—C6	−44.1 (4)	C6—N1—C10—C9	1.0 (5)
C2—W1—N1—C6	44.0 (4)	W1—N1—C10—C9	−178.9 (2)
C4—W1—N1—C6	−131.8 (4)	C8—C9—C10—N1	−0.3 (5)
C5—W1—N1—C6	134.8 (4)	C11—C9—C10—N1	176.5 (3)
C1—W1—C2—O2	71 (4)	C15—N2—C11—C9	−69.3 (3)
C3—W1—C2—O2	−18 (4)	C14—N2—C11—C9	168.8 (3)
C4—W1—C2—O2	2(7)	C15—N2—C11—C12	168.6 (3)
C5—W1—C2—O2	160 (4)	C14—N2—C11—C12	46.7 (3)
N1—W1—C2—O2	−113 (4)	C10—C9—C11—N2	152.7 (3)
C1—W1—C3—O3	−7(10)	C8—C9—C11—N2	−30.6 (5)
C2—W1—C3—O3	84 (10)	C10—C9—C11—C12	−92.4 (4)
C4—W1—C3—O3	−94 (10)	C8—C9—C11—C12	84.2 (4)
C5—W1—C3—O3	30 (13)	N2—C11—C12—C13	−35.7 (3)
N1—W1—C3—O3	176 (10)	C9—C11—C12—C13	−157.3 (3)
C1—W1—C4—O4	−71 (5)	C11—C12—C13—C14	12.4 (4)
C3—W1—C4—O4	19 (5)	C15—N2—C14—C13	−162.2 (3)
C2—W1—C4—O4	−1(8)	C11—N2—C14—C13	−39.3 (3)
C5—W1—C4—O4	−159 (5)	C12—C13—C14—N2	15.9 (4)
N1—W1—C4—O4	113 (5)		
